# Self-Leadership and Innovative Behavior: Mediation of Informal Learning and Moderation of Social Capital

**DOI:** 10.3390/bs12110443

**Published:** 2022-11-11

**Authors:** Hyesun Kang, Minyoung Song, Yiran Li

**Affiliations:** 1 School of Management, Kyunghee University, 701, Orbis Hall, 24, Kyungheedae-ro, Dongdaemun-gu, Seoul 02453, Korea; 2 Korea Labor and Employment Service, Mapo-Daero 130, Mapo-gu, Seoul 04212, Korea; 3 Institute for Educational Research, Faculty of Education, Yonsei University, 50, Yonsei-ro, Seodaemun-gu, Seoul 03722, Korea

**Keywords:** self-leadership, innovative behavior, informal learning, social capital, organizational innovation, PROCESS Macro

## Abstract

As the business environment is rapidly changing, interest in the innovation of organizational members is accelerating. Therefore, this study investigated how individual-level resources, particularly self-leadership, affect workers’ innovative behavior. Many studies have emphasized that employee initiative can lead to job performance at the individual level and organizational performance improvement. Self-leadership is a spontaneous and an active behavior, or mindset, defined as the ability to lead an individual in challenging situations characterized by learned behaviors that can be augmented by training. It is of interest to many researchers and practitioners. Further, we tested the mediation of informal learning, another individual-level resource, in this relationship and the moderation of social capital, a social resource, in the mediation. We analyzed the responses of 551 employees of South Korean companies using Model 6 and 14 of PROCESS Macro. The results revealed that self-leadership positively influenced workers’ innovative behavior, and informal learning mediated this relationship. We also confirmed that social capital strengthened the positive mediating effect of informal learning. This study empirically verifies the role of self-leadership, informal learning, and social capital as the determinants of innovative behavior and expands the discussion on leadership by highlighting the significance of self-leadership as opposed to traditional leadership approaches.

## 1. Introduction

As the business environment is inherently uncertain, organizations must constantly adapt to the changing landscape, which they often do through innovation. Consequently, organizational innovation has caught the attention of organizational stakeholders and researchers [1,2,3]. With growing emphasis on organizational innovation, interest is also rising in the innovative behavior of organizational members [4,5,6,7]. Bateman and Crant [8] defined individual innovative behavior as the effort of creating a new environment or changing the existing environment at an individual worker’s level. Crant [9] defined it as an individual proactively creating a new situation rather than passively adapting to the existing one. Innovative behavior differs from creativity because the former involves not only proposing a new and beneficial idea, but also implementing it [10]. As innovative behavior involves actions that can improve an organization’s functioning, it directly contributes to the organization’s survival [9].

Previous studies have shown that the innovative behavior of organizational members can be a source of competitive advantage for the organization [7]. They have also presented the determinants of members’ innovative behavior. For instance, Martins and Terblanche [11] stated that organizational culture can be a crucial antecedent. Workers perceive innovative behavior as highly desirable and actively perform it when the organizational culture values the development of new products and processes. To determine how leadership influences workers’ innovative behavior, Elenkov and Manev [12] analyzed the data from 12 European countries and found that innovative behavior can be promoted in a sociocultural context that rewards workers for it. Jung, Chow, and Wu [13] found that transformational leadership can boost innovative behavior. They explained that transformational leadership changes workers’ value systems, motivates them to achieve higher performance levels, and stimulates them to think creatively. Overall, studies that investigated the determinants of workers’ innovative behavior have established that mainly external rewards and influences induce workers to engage in innovative behavior.

In contrast, studies that explored individual innovative behavior have stressed that this behavior is primarily determined by the individual’s mindset and the motivational processes behind wanting to implement change [9,14]. Innovative behavior involves generating and implementing new ideas. However, it is difficult to predict the obstacles that will arise in engaging in this behavior, thus making it quite challenging to maintain the behavior until the desired result is achieved [15]. Therefore, considering that behavior can be self-controlled to adapt to a changing environment, it is easier to engage in innovative behavior when it is managed internally. Moreover, researchers have asserted that internal regulatory processes have a stronger influence on the maintenance of behaviors than external influences [9,14]. Therefore, it may be more effective to boost innovative behavior internally instead of relying on external sources.

Previous studies have shown how individual characteristics influence innovative behavior [16,17]. For example, workers with high self-esteem tend to learn or seek change [18], extroversion is associated with innovative behavior [19], and positive people are highly likely to engage in innovative behavior [20]. However, individual traits such as personality, core self-evaluation, and positivity are generally genetically determined. Thus, a person can boost innovative behavior to a limited extent by managing these traits. Accordingly, this study investigates the influence of self-leadership on innovative behavior. Self-leadership is a voluntary and proactive behavior or a way of thinking, defined as the tendency or ability of individuals to lead themselves in challenging situations [21]. Manz [22] explained that it can be conceptualized as a learned behavior; it can be trained and cultivated.

Based on extant theories and research, this study posits and verifies hypotheses on the mediating and moderating factors in the relationship between self-leadership and innovative behavior. More specifically, we investigate how individual-level resources, particularly self-leadership, affect innovative behavior. We also test whether informal learning (another individual-level resource) mediates the relationship between self-leadership and innovative behavior, and whether social capital (a social resource) moderates this mediation.

Self-leadership enables individuals to respond appropriately to new learning needs that arise during innovation activities. Specifically, the mechanism of the relationship between self-leadership and innovative behavior can be explained as an effective learning process. Previous studies have revealed the positive impact of learning on innovative behavior [23,24]. However, in most cases the focus was on the effectiveness of formal learning that was structurally designed to deliver formal content [25]. Informal learning, commonly known as spontaneous and introspective learning, is distinguished from formal learning in that it is characterized by observation and imitation, collaboration and communication, and personal reflection, that is, social constructivist learning.

In the process of informal learning, individuals actively interact with the social environment in terms of actively seeking feedback and advice and sharing work experiences [26]. This behavior aids the innovation process by enabling individuals to acquire knowledge and reduce potential errors. The model of this study—which explains the relationship between self-leadership and innovative behavior as the mediating effect of informal learning and the moderating effect of social capital—is based on social cognitive theory. Social cognitive theory explains that human behavior can be induced by the interaction of internal and external influencers [27]. According to social cognitive theory, an individual can increase self-efficacy for a specific task through interaction with the social environment, which leads to improved learning performance and beneficial task behavior [28]. Accordingly, this study assumes that the effect of informal learning carried out through self-leadership can be stronger in a situation where social capital is built high.

Overall, this study demonstrates that self-leadership regulates intrinsic rewards, thus increasing workers’ motivation to achieve goals and willingness to learn informally. The augmented knowledge of workers results in more innovative behavior, which can be further strengthened if social capital exerts an impact on this relationship.

## 2. Theory and Hypotheses

### 2.1. Self-Leadership

External motivational factors influence an individual’s behavioral intentions through internal processes. Thus, internal processes are ultimately the key to influencing behavioral intentions. Manz [22] held that as organizations employ different control systems to influence the behavior of members, members also use internal standards and evaluation systems to manage their behavior. Therefore, there is a process through which members influence themselves, which considerably affects their final behavior. Manz [22] expanded this concept of a self-management process and introduced a self-influence process called self-leadership. Self-leadership is the process through which individuals use specific behavioral and cognitive strategies to influence and guide themselves [22]. It is rooted in the self-control theory and is a leadership substitute according to Kerr and Jermier [29]. Alternatively, self-leadership is a state in which individuals lead the motivational process themselves with a great desire to achieve a goal and direct their behavior and capability toward it.

Studies have shown that self-leadership positively impacts organizational performance such as improving productivity, facilitating a successful career, increasing job satisfaction, and reducing absenteeism. It also enhances individual well-being by reducing stress and anxiety and increasing self-efficacy [30,31,32,33,34]. It facilitates the maintenance of desirable behaviors even without external control, thus contributing to the job success of individuals. Moreover, it emphasizes individuals’ intrinsic satisfaction with their actions and helps them in maintaining a positive inner state while performing actions.

**Figure 1:**
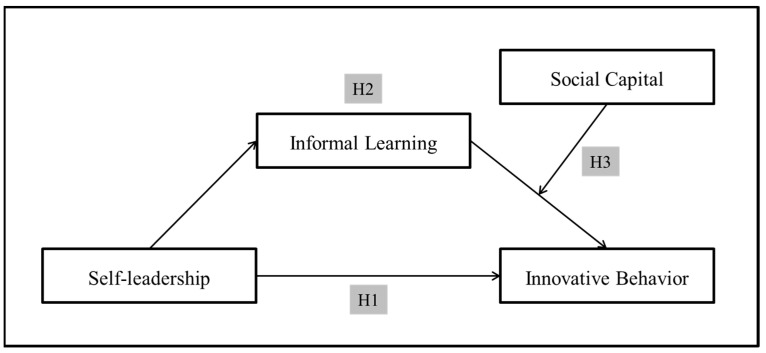
Research model.

Researchers have mainly cited intrinsic motivation as the mechanism through which self-leadership affects performance [21,35]. Many have stressed the influence of intrinsic rewards in the motivational process for achieving specific goals. Using the self-determination theory, Deci and Ryan [36] stated that motivation can be the most impactful driver as intrinsic rewards satisfy an individual’s need for confirming self-competence and self-determination. In self-leadership, influential strategies raise the perceived intrinsic reward of the current task, thus increasing the strength and duration of efforts to achieve the goal [37]. Self-leadership can also promote innovative behavior. Specifically, it does so by influencing the use of individual learning strategies and activating informal learning in the process of self-leadership. This informal learning can also be more effective when social capital is well established. Social learning theory avers that individuals interact with their social environment and can enhance their learning influencers [27]. Accordingly, this study established the following research model (Figure 1).

### 2.2. Self-Leadership and Innovative Behavior

In addition to task performance, the mechanisms of self-leadership can also be applied to innovative behavior. Studies have demonstrated a positive relationship between motivation and innovation [38,39]. If individuals are offered extrinsic rewards for innovative performance, they can be motivated to behave based on the reward offered [40]. However, extrinsic rewards can weaken intrinsic motivation over time [41]. Numerous researchers have stressed that intrinsic rewards are more motivating than extrinsic ones. For instance, Amabile [42,43] held that intrinsic motivational factors made individuals process information more actively than extrinsic motivational factors. Thus, the former results in in-depth information processing and, ultimately, the generation of high-quality solutions or novel problem-solving approaches. Hammond et al. [44] found that intrinsic motivation had a strong and highly consistent relationship with innovation. Howell [45] stated that personal traits and behaviors of individuals who successfully developed and applied innovative ideas could be utilized to boost innovative behavior. Meanwhile, Phelan and Young [46] found that self-leadership increases individual innovative behavior. Researchers have defined self-leadership as a reflective internal process through which individuals consciously and constructively explore their thoughts and intentions to implement desired changes. Moreover, they have stated that individuals can improve and innovate their tasks using this process.

Workers’ innovative behavior is defined as the generation and implementation of new ideas at the individual level [47]. Scott and Bruce [48] believed that workplace innovation at the individual level is a behavioral process comprising three steps. In the first step, individuals recognize a problem and present or adopt novel solutions and ideas. Subsequently, they promote their ideas or work to build legitimacy inside and outside the organization. Finally, they produce a prototype suitable for the organization to realize the novel idea. Hence, innovative behavior can be considered a multi-step process which consists of individuals recognizing the need for a new idea, promoting and building support for the idea, and ultimately making it suitable for the organization. Innovative behavior does not end at the generation of a new idea. The idea must be accepted by others to be applied in the organization. Therefore, innovative behavior requires more effort than creativity [43], which centers around generating new and useful ideas [10,48]. Among the stages of innovative behavior, the implementation stage, particularly, can pose several obstacles, such as resistance to change. Therefore, innovative behavior requires more work to maintain efforts than performing general tasks [49]. Innovative behavior is more likely to occur under self-leadership because engaging in innovative behavior demands a stronger motivation than performing general tasks. Self-leadership can provide strong motivational processes.

Based on these arguments, we formulated the following hypothesis.

**Hypothesis 1** **(H1).** *Self-leadership will increase workers’ innovative behavior.*

### 2.3. Mediation of Informal Learning

The mechanism through which self-leadership increases workers’ innovative behavior can be explained by the extent of knowledge available for innovation. Hammond et al. [44] stated that individuals find cognitive sources of obtaining new ideas using their accumulated knowledge and experience. Previous studies have also shown that a salient prerequisite of innovation is the availability of resources or the possibility of obtaining resources by learning [50,51].

Thus, we can say that the volume of available knowledge can be increased by learning. Workplace learning is generally classified into formal and informal learning [52,53]. Formal learning can be regarded as a traditional educational approach, with interaction as the standard learning method [54]. It consists of planned activities that are designed to help organizational members acquire job-related skills and knowledge. Nearly all educational and developmental programs that an institution provides fall into this category. Formal learning is more systematic than informal learning and is generally conducted in an educational environment, such as a classroom [55]. Conversely, informal learning is learner-led and depends on the learner’s needs. It occurs through activities such as self-reflection and observation and is generally driven by the learner’s motivation to progress [56,57].

Researchers have reported that most processes of obtaining new knowledge in the workplace take place informally [58]. For instance, Bear et al. [59] reported that 75% of learning within organizations is informal learning. Informal learning is continuous and driven by the individual’s motivation to learn [60]. Thus, several researchers have asserted that it can overcome the limitations of formal learning programs and significantly contribute to improving learners’ future performance cf. [61]. Enos, Kehrhahn, and Bell [62] explained that informal learning led by actions and reflection facilitates appropriate behavior in response to changing situations, thus enabling individuals to achieve high performance.

As innovative behavior necessitates the exploration of new ideas, it requires more contextual and informal learning than pre-designed formal learning. It needs a deep knowledge of the target domain and an extensive knowledge of the adjacent ones [15,63]. Having holistic knowledge is beneficial because individuals use different types of knowledge and skills to engage in innovative behavior. Moreover, they must perform actions and review their suitability simultaneously, and informal learning can be helpful [62]. As formal learning is pre-designed, the scope of learning and acquiring knowledge and skills is more limited than in informal learning, which provides an unlimited scope of learning [61]. Furthermore, informal learning is more effective in innovative problem-solving because it is difficult to ascertain which domain knowledge will be required to solve future problems. In informal learning, domain knowledge can be flexibly adjusted based on the problem at hand.

Given that new knowledge must be obtained to generate and implement new ideas, studies have shown that active learning influences innovativeness [64,65]. For instance, Park et al. [66] argued that workers’ continuous efforts to acquire relevant skills and knowledge is the key to innovative outcomes. Similarly, Rhee, Park, and Lee [67] stated that individuals’ continued commitment to learning is the key to innovation. This motivation to learn actively is related to self-leadership. Individuals who are strong self-leaders are highly motivated to achieve their goals. Thus, they are extremely motivated to acquire knowledge for problem-solving [68]. These individuals do not rely solely on formal learning; they also engage in informal learning [69].

In self-leadership, individuals self-regulate their behavior and utilize intrinsic rewards to achieve goals. Informal learning can occur naturally in this process. Behavior regulation strategies mainly involve the recognition of standards and behavioral regulation [70]. Therefore, individuals must acquire information and assess circumstances, which can result in informal learning. When it comes to utilizing intrinsic rewards to exhibit a specific behavior, individuals must first identify desirability in their actions [21]. This can lead to continuous reflection and social exploration (or informal learning). Thus, informal learning is a continuous process for self-leading individuals, and we can infer that these individuals will exhibit more innovative behavior using the cognitive resources they acquire from informal learning.

Accordingly, we formulated the following hypothesis.

**Hypothesis 2** **(H2).** *Informal learning will mediate the effect of self-leadership on workers’ innovative behavior.*

### 2.4. Moderation of Social Capital

To implement a new idea, individuals must share their idea with others in the organization. Innovation can be considered a social process because it not only encompasses the generation of ideas, but also their implementation [71]. Drazin, Glynn, and Kazanjian [72] stated that the process of achieving innovative outcomes is inherently multi-level. Individuals first generate an idea. Then, they discuss it with others to receive feedback. Based on the feedback, they revise the idea to achieve innovative outcomes. Axtell et al. [73] state that most jobs in an organization are inherently interdependent, and the same interdependence applies to workers’ innovations. Even if workers are able to innovate themselves, they require support and approval from others and the opportunity to implement the innovation [74]. Therefore, social and contextual factors play a crucial role in workers’ innovative behavior.

Studies investigating the antecedents of innovative behavior have emphasized the role of learning [66,75,76]. However, learning concerns only the accumulation of knowledge. Considering that innovative behavior involves not only the generation of ideas, but also their acceptance and promotion, we can infer that social capital is as crucial as knowledge capital for workers’ innovative behavior [77,78]. Social capital is an intangible capital formed through reciprocal relationships between members [79]. It comprises three dimensions: structural, relational, and cognitive social capital. The structural dimension indicates the connectivity between workers, the frequency of contact, and the closeness of relationships [80]. The relational dimension represents the relationships that develop between workers as they interact and the quality of these interpersonal relationships [81]. The cognitive dimension refers to the cognitive system shared by members of a network or organization, consisting of shared values, language, vision, and culture [79,82].

Individuals who have structural social capital can access actors from various domains relatively easily, having ample opportunities to access and converge diverse knowledge [83]. Consequently, they can enhance their informal learning. With relational social capital, individuals can gain respect and acceptance from their peers. It can improve their competence to innovate and broaden their behavioral repertoire, thus increasing participation in innovative work behavior [84]. Moreover, forming high-quality positive relationships with their colleagues provides them a sense of safety when they attempt to change or improve work processes, thus facilitating the occurrence of more innovative behavior [85]. The cognitive dimension helps individuals adopt behaviors and practices that are considered desirable based on the common values and norms [86]. It suggests that as the cognitive social capital increases, it becomes easier for individuals to implement ideas in the organization.

We can say that self-leadership and informal learning, the main variables in this study, are the determinants of workers’ innovative behavior. However, social capital can also be considered a determinant because informal learning is learning socially. Moreover, innovative behavior not only involves generating new ideas, but also spreading them throughout the organization. Thus, we can infer that as social capital increases, the positive effect of informal learning on innovative behavior becomes stronger.

Accordingly, we formulated the following hypothesis.

**Hypothesis 3** **(H3).** *Social capital will moderate the mediation of informal learning in the positive relationship between self-leadership and innovative behavior, such that it strengthens the mediating effect of informal learning.*

## 3. Methodology

### 3.1. Sample

We conducted a self-report study on employees from 21 South Korean companies. This group of companies comprised small, medium-sized, middle-standing, and large enterprises, as well as public institutions in the finance, distribution, computer, education, and service industries. To control for industry effects naturally, we included enterprises operating in different industries in our sample. Data were collected over 17 days from 19 August 2019 to 4 September 2019. A total of 650 questionnaires were distributed, and 583 valid responses were recovered, making the response rate 89.7%. We excluded 32 insincere responses, which were incomplete, had identical answers across a certain section, or had answers missing for some items. Thus, 551 responses were used in our analysis. We conducted a preliminary power analysis using G*Power3. It revealed that the sample size must be at least 129 if there are 4 measurement variables, 1 independent variable, 1 mediator, and 1 dependent variable. As our sample’s size is 551, it can be considered to have high statistical power [87].

### 3.2. Frequency Analysis

The results of the frequency analysis revealed that the percentage of males (74.6%) was higher than that of females in our sample, thus indicating that the percentage of male employees is generally high in Korean companies. Most respondents were in their 30s (39.2%), followed by those in their 40s (38.3%) and 50s (12.3%). Those in their 20s (10.2%) were the least in number. It is probably because employment difficulties and economic stagnation delay the start of work life for college graduates, and they start working at a later age. Most respondents were university graduates (73.7%), followed by those who were graduate school graduates (17.4%), technical college graduates (5.8%), and high school graduates (3.1%). The high percentage of university graduates suggests that workplaces generally have a huge number of university graduates. Most respondents were team leaders, managers, or deputy general managers (53.7%), and senior staff or assistant managers (33.4%). The remaining respondents were general staff (7.1%), general or branch managers (4.7%), and executives or at a higher position (1.1%). These percentages are similar to the general composition of an organization, with team leaders, managers, or deputy general managers forming the largest group of employees. Furthermore, 8.0% of the respondents had been working at the company for less than a year, 17.4% for 1–5 years, 24.3% for 5–10 years, 17.8% for 10–15 years, 11.8% for 15–20 years, and 20.7% for 20 or more years. Most respondents were part of the company’s management (66.1%). The remaining worked in the R&D (10.5%), sales (10.0%), service (3.8%), production or technical (4.7%), and other departments (4.9%). Moreover, 52.5% worked in the finance or insurance industry, 9.8% in the information or telecommunications industry, 8.3% in the distribution or sales industry, 6.0% in the manufacturing industry, 4.9% in the education industry, 4.0% in the service industry, 0.9% in the construction industry, and 12.2% in other industries. The remaining respondents were civil servants. Moreover, 17.4% respondents were contract workers for a fixed term. The rest were permanent employees.

### 3.3. Measurement

To measure the four measurement variables, we selected four measurement scales that were found to be reliable and valid in previous studies. All variables except the control variables were measured using a 5-point Likert scale, with 1 denoting “Not at all”, 3 expressing “Neutral”, and 5 denoting “Very much”.

#### 3.3.1. Innovative Behavior

Following Scott and Bruce [48] and Janssen [88], innovative behavior was operationally defined as the degree to which individuals intentionally create, introduce, and apply new ideas that help enhance their performance in task role, group, or organization. We modified Scott and Bruce’s [48] measurement scale to measure this variable, using six items, including “I seek new technologies, approaches, and ideas related to my work”.

#### 3.3.2. Self-Leadership

Based on the definitions given by Prussia, Anderson, and Manz [89], Houghton and Neck [90], and Neck and Houghton [21], we defined self-leadership as the degree to which individuals influence themselves to behave and perform in desirable ways. We modified Prussia, Anderson, and Manz’s [89] measurement scale and divided self-leadership into three dimensions: behavior-focused strategies, natural reward strategies, and constructive thought pattern strategies. Behavior-focused strategies were measured using six items, including “I frequently check my work progress”. Natural reward strategies were measured using six items, including “I strive to expand my role at work”. Constructive thought pattern strategies were measured using eight items, such as “I use my own approach to solve problems”.

#### 3.3.3. Informal Learning

We defined informal learning as those learning activities in which an individual acquires knowledge and skills through physical, cognitive, and emotional efforts in the workplace along with the formal education the organization provides [91,92]. We modified the measurement scale used by Choi [92] and Choi and Jacobs [93] based on this study’s purpose. A total of 15 items were used to measure informal learning, with 5 items to measure the exploration of learning opportunities, 6 to measure learning with others, and 4 to measure reflection.

#### 3.3.4. Social Capital

Social capital was operationally defined as the degree of available relationship networks and the capital embedded in an individual’s relationship network [79]. To measure this variable, we modified Van den Hooff and Huysman’s [94] measurement scale and constructed 16 items. Structural social capital was measured using 7 items, including “My colleagues know what kind of knowledge is required to perform my job”. Cognitive social capital was measured using 4 items, including “My colleagues and I use the same technical language”. Relational social capital was measured using 5 items, including “I believe that my colleagues and I are on the same side”.

#### 3.3.5. Data Analysis Procedure

We used SPSS 27.0, Amos 28.0, and PROCESS Macro (Nos. 6 and 14) developed by Hayes [95] to verify the measurement, structural, and moderated mediation models. These statistical programs are used in the social science field. Our procedure comprised the following steps. First, we conducted a frequency analysis to determine our sample’s demographics in terms of sex, age, educational background, position, tenure, department, industry, and employment type. Second, we performed a confirmatory factor analysis based on the variables’ operational definition and measurement tools suggested in previous studies. Then, we verified internal consistency and content validity. Third, we conducted Harman’s single factor test as a post hoc test to check common method bias because we collected data from a single source. Fourth, we analyzed convergent and divergent validities and tested the structural model. Finally, we tested our research hypotheses. Unlike the existing methods of analyzing mediating effects, PROCESS Macro can analyze multiple mediators and moderators at a time, reflect measurement errors in the research model, and statistically verify individual mediating effects [95]. The estimation of effects in regression analysis tends to be biased compared to that in structural equation modeling due to random measurement errors [96]. However, a comparative study by Hayes, Montoya, and Rockwood [97] found no difference in the estimated coefficient values when using ordinary least squares (OLS) regression-based path analysis and the structural equation modeling program, even in small samples. There was a difference in the standard error. However, it was expected because the sample variance estimations of the OLS and maximum likelihood estimation methods were based on different statistical assumptions. Therefore, it can be considered a non-issue. This study investigated mediation and moderated mediation effects between factors related to workers’ innovative behavior, rather than measuring the overall structural suitability of the research model. Therefore, we used PROCESS to test our hypotheses.

## 4. Results

### 4.1. Measurement of Model

Table 1 presents the results of testing the reliability of our research variables. The overall Cronbach’s alpha of self-leadership, the independent variable, and its sub-elements was at least 0.600. Similarly, overall Cronbach’s alpha of informal learning, the mediator, and its sub-elements was at least 0.600. We removed items 19 and 20 that measured reflection, a sub-element of informal learning, because their factor load was less than 0.600. The value of Cronbach’s alpha of our dependent variable, innovative behavior, was at least 0.600. Finally, the value of Cronbach’s alpha of all items of social capital was at least 0.600, except for items 9 and 11 on cognitive social capital. After excluding these two items, Cronbach’s alpha of the items of structural, cognitive, and relational social capital was at least 0.600. To conclude, the value of Cronbach’s alpha of the observation items of each research variable ranged between 0.610 and 0.900, indicating a high level of reliability.

As stated earlier, the possibility of common method bias cannot be completely avoided in this study. An exploratory factor analysis with the factor set to one explained 30.87% of the total variance. Moreover, all observation variables were set as one latent factor in the confirmatory factor analysis. It showed a substantially lower fit than that of the measurement model ($x^{2}$ = 1557.652, df = 90, CMIN/DF = 17.307, GFI = 0.595, AGFI = 0.460, CFI = 0.696, NFI = 0.684, IFI = 0.696, TLI = 0.645, RMSEA = 0.172, RMR = 0.064). These results indicate that errors arising from common method bias are not severe enough to impact our results [98].

We checked for convergent validity to investigate the relationship between the observation items and latent variables of this study. Table 2 shows the results. The standardized coefficient of self-leadership, informal learning, innovative behavior, and social capital was at least 0.500 after excluding items 9 and 11 on cognitive social capital and items 19 and 20 on reflection. This result verified the construct and content validity of each observation item. Convergent validity (secondary factor analysis) was also satisfied since the standardized coefficient of all observation items was at least 0.500.

Subsequently, we checked the reliability, composite reliability (CR), average variance extracted (AVE), convergent validity, and discriminant validity of our variables. Our independent variable (self-leadership) and mediators (informal learning and social capital) have a second-order factor structure. Therefore, we performed a second-order confirmatory factor analysis, and the results are shown in Table 2. The results of the goodness-of-fit indices indicate that absolute, incremental, and parsimonious fit indices of the structural equation model satisfy the criteria of acceptance. Reliability was judged based on CR and AVE, as proposed by Fornell and Larcker [99]. Reliability is established if the AVE is at least 0.500 and CR at least 0.700 [99]. The AVE of our variables was at least 0.500, and the value of CR was at least 0.800, indicating that the two conditions for construct reliability were satisfied. Thus, we verified internal consistency, construct reliability, and convergent validity, confirming the validity of our research model.

Before testing our hypotheses, we performed an analysis to determine correlation and discriminant validity between the constructs. Table 3 presents the results. Self-leadership was significantly correlated to informal learning (r = 0.665), innovative behavior (r = 0.608), and social capital (r = 0.612). Informal learning was significantly correlated to innovative behavior (r = 0.490) and social capital (r = 0.659). The correlation between innovative behavior and social capital (r = 0.344) was also high.

To verify discriminant validity, we compared the constructs’ AVE and the square of the correlation coefficient, following Fornell and Larcker [99]. Discriminant validity is confirmed if AVE is greater than the square of the correlation coefficient [99]. Table 3 reveals that the largest correlation coefficient between the variables is 0.665 (correlation between self-leadership and informal learning) and its square is 0.442. The smallest AVE (0.660) is larger than this number, confirming discriminant validity.

### 4.2. Hypotheses Testing

We used Model 6 of PROCESS Macro, proposed by Hayes, to verify the mediating effect of informal learning on the relationship between self-leadership and innovative behavior [95]. Table 4 presents the results. Evidently, the model was statistically significant (F = 62.4799, R2 = 0.446, *p* < 0.000). Self-leadership significantly and positively influenced informal learning (β = 0.644, *p* < 0.001) and innovative behavior (β = 0. 650, *p* < 0.001). Furthermore, informal learning significantly and positively affected innovative behavior (β = 0.226, *p* < 0.001).

Subsequently, we extracted 5000 samples using the bias-corrected bootstrapping method to verify the mediating effect. Table 5 presents its results. The bias-corrected approach reflects asymmetry in bootstrap estimates more strictly to determine the validity of the mediating model using the upper and lower limits of the confidence interval. This method can yield more accurate results when distribution of bootstrap estimates is skewed [95]. For an indirect effect to be statistically significant in bootstrapping, zero should not be present in the 95% confidence interval [95].

The PROCESS Macro bootstrapping results for self-leadership → informal learning → innovative behavior showed that the direct effect of self-leadership on innovative behavior (β = 0.650, *p* < 0.05) was significant. The indirect effect of self-leadership on innovation behavior through informal learning (β = 0.146, *p* < 0.001) was also significant. Furthermore, the lower and upper limits of this indirect effect were 0.062 and 0.230, indicating that 0 was not present. This result indicates that informal learning significantly mediates the relationship between self-leadership and innovative behavior, supporting Hypotheses 1 and 2.

Previous studies analyzed only two groups (a high and low group) to verify moderated mediation. However, using PROCESS Macro for a conditional indirect effect analysis, proposed by Hayes [95], makes it possible to compare three groups: high (Mean + 1SD), medium (Mean), and low (Mean-1SD). Hence, differences between the value and significance of moderated mediation in each group can be investigated. Hayes [95] explained that when an indirect effect is conditional on a moderator, there exists not just one single indirect effect. Thus, individuals must conditionalize the indirect effect at different values of the moderator. This signifies that even if the moderated mediating effect is rejected, it may differ between groups based on the moderator’s level, which must therefore be examined. Therefore, we investigated both the conditional indirect effect and the moderation of social capital on the mediation of informal learning. We performed an analysis using the bias-corrected bootstrapping method through Model 14 of PROCESS Macro to test whether social capital moderated self-leadership → informal learning → innovative behavior, the relationship between informal learning and innovative behavior, and the relationship between self-leadership and innovative behavior. For the effect to be statistically significant, 0 should not be present between the lower limit confidence interval (LLCI) and upper limit confidence interval (ULCI) of the 95% confidence interval. Additionally, the variables self-leadership, informal learning, and social capital were mean-centered to avoid potential multi-collinearity between the independent variables or moderators and interaction terms. Innovative behavior did not require mean centering, and thus it was used without modification.

To verify the moderated mediating effect, we input self-leadership as the independent variable, innovative behavior as the dependent variable, informal learning as the mediator, and social capital as the moderator based on Hayes’ Model 14 in PROCESS Macro [95]. An analysis of 551 responses with a 95% confidence interval as given in Table 6 reveal that self-leadership positively affects informal learning (t = 19.146, *p* < 0.001). The interaction of informal learning and social capital significantly affects innovative behavior (t = 3.046, *p* < 0.002). This result demonstrates that self-leadership significantly influences workers’ innovative behavior through informal learning, and this social capital moderates this relationship.

Table 7 presents the results of testing the conditional indirect effect of social capital on the influence of informal learning on innovative behavior. The moderated mediating effect of social capital was statistically significant (LLCI = 0.032, ULCI = 0.198). More specifically, the mediating effect was β = 0.153 when social capital was low (LLCI = 0.056, ULCI = 0.250). As social capital rose to the medium level (LLCI = 0.119, ULCI = 0.306), its mediation increased to β = 0.212. This effect became stronger (β = 0.270) when social capital reached the highest point (LLCI = 0.164, ULCI = 0.377). Therefore, Hypothesis 3 was supported. Moreover, zero was not present between the LLCI and ULCI in the low and medium groups, indicating statistical significance.

**Figure 2:**
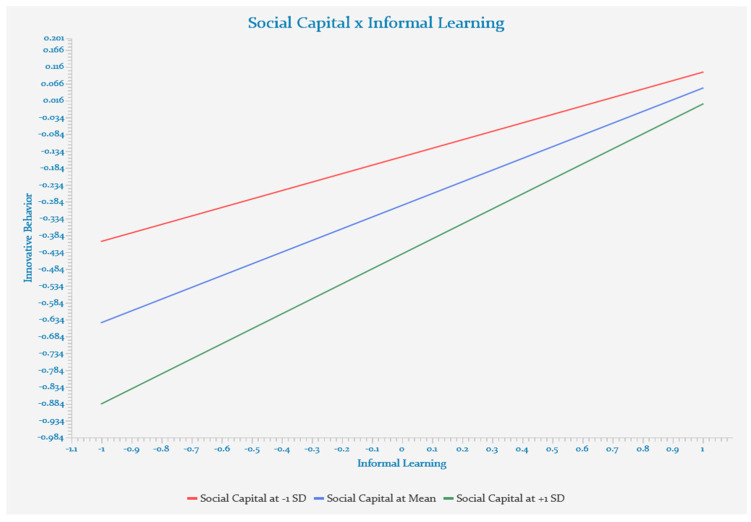
Moderated mediating effect of social capital.

Figure 2 illustrates the conditional indirect effect of social capital on the impact of self-leadership on innovative behavior through informal learning. The moderated mediating effect of social capital was statistically significant (LLCI = 0.032, ULCI = 0.198). More specifically, the mediating effect was β = 3.145 when social capital was low (LLCI = 0.056, ULCI = 0.250). As social capital rose to the medium level (LLCI = 0.119, ULCI = 0.306), the mediating effect was statistically significant at β = 3.920. When social capital rose to the highest level (LLCI = 0.164, ULCI = 0.377), its effect was strengthened (β = 0.424). Therefore, the moderated mediating effect of social capital was statistically significant in the impact of self-leadership on innovative behavior through informal learning.

## 5. Discussion

### 5.1. Conclusions

This study investigated the relationship between self-leadership and innovative behavior, the mediation of informal learning, and the moderation of social capital in the mediation of informal learning. The results are summarized as follows.

First, the more individuals engage in self-leadership, the more innovative behavior they exhibit. Innovative behavior requires a stronger motivation level than normal task performance. Therefore, it is more likely to occur in self-leadership situations that can lead to stronger motivational processes. The results of this study empirically prove that self-leadership can contribute to the innovative behavior of workers. Second, informal learning positively mediates the relationship between self-leadership and innovative behavior. Individuals who practice self-leadership are constantly learning, which means that informal learning occurs at a high frequency. Moreover, through this informal learning, they have more cognitive resources, which, in turn, leads to further innovative behaviors. The results of this study are consistent with those of previous research that established that self-leadership can increase achievement levels by self-directing internal motivational processes toward achieving a goal [100,101]. Third, social capital strengthens the mediation of informal learning. A key characteristic of informal learning is that interaction with the social environment occurs frequently in the learning process. Considering that innovative behavior involves the diffusion and application of ideas, the effectiveness of informal learning can be maximized through the construction and reinforcement of social capital. Social capital can be considered an effective social determinant of innovative behavior because it strengthens the influence of self-leadership and informal learning on innovative behavior. This result is consistent with those of previous studies that emphasize the influence of social capital on an individual’s positive behavior in an organization [102,103].

In summary, these empirical results demonstrate that self-leadership regulates intrinsic rewards for achieving goals, thus increasing motivation and informal learning. Increased knowledge ultimately results in more innovative behavior, and this effect can be reinforced by social capital.

### 5.2. Theoretical Implications

The results of this study present the following implications. First, it empirically proves that self-leadership, informal learning, and social capital are the determinants of innovative behavior. Second, it furthers the discussion on leadership by emphasizing how workers influence and lead themselves, as opposed to conventional leadership styles such as charismatic or transformational leadership. DiLiello and Houghton [100] argued that while traditional top-down leadership styles were appropriate in the past, focus must now be shifted to self-leadership. Third, many researchers have stated that it is difficult to maintain workers’ motivation when only traditional leadership approaches are utilized [104,105]. In such situations, this study proves useful because it confirms the role of self-leadership as an alternative to traditional leadership approaches. Fourth, we find that social capital enhances the impact of informal learning on workers’ innovative behavior. The relationship between workers’ self-leadership and innovative behavior is contingent on their social capital. Numerous researchers have agreed that social capital is a relational resource present in an individual’s social environment [106]. This study reinforces the claims of previous studies that social capital contributes to creating a close network of members who interact and collaborate with each other, thereby enabling effective work performance. Finally, it reveals that individual-level innovative behavior can be induced and reinforced through the combination of their self-leadership, informal learning, and social capital. It asserts the positive effect of integrating three different antecedents on innovative behavior, thus extending the literature on the topic. Previous studies investigated the influence of self-regulation, intrinsic motivation, and social capital on innovative behavior. However, they separately explored the impact of resources or individual characteristics cf. [107,108].

### 5.3. Practical Implications

The results of this study have the following practical implications. First, owing to changes in the organizational environment, new aspects such as diversity, delegation, and horizontal relationships are emerging in the discussion on desirable leadership styles cf. [109]. Researchers have claimed that as the values of organizational members change rapidly, it becomes difficult to achieve organizational goals using only traditional leadership approaches relying solely on the leader. Therefore, we believe that organizations must focus on self-leadership because workers actively obtain knowledge and skills required for innovative behavior using behavior-focused and constructive thought pattern strategies. Second, as self-leadership motivates an individual to achieve goals using intrinsic rewards and self-determination, it can simultaneously improve performance and well-being [110]. Organizations should train workers to practice self-leadership and form a culture of self-directed behavior. Third, as social approval is critical for the implementation and promotion of workers’ innovative behavior, efforts to build social capital among organizational members are essential. Fourth, organizations should appreciate and reward workers’ innovative endeavors. A fair system that rewards workers for their contributions may encourage them to perform more innovative behavior. Finally, organizations must focus on creating a sense of security for workers, even when they attempt to deviate from norms at the organizational and team levels.

### 5.4. Limitations and Directions for Future Research

Despite its utility, this study has some limitations, which serve as directions for future research. First, it investigated social capital as a social variable influencing informal learning at the individual level. However, Grossman and Salas [110] stressed that the transfer of learning outcomes can be strengthened by factors not only at the individual level, but also at the environmental level, such as organizational climate. Therefore, future studies must investigate other moderators as well. Second, we estimated causal relationships based on a cross-sectional study rather than a time series or experimental study. This research approach may determine causal relationships of social phenomena inaccurately. Social and psychological domains emphasize that cognition, emotion, attitude, and behavior patterns change over time. Therefore, researchers must consider conducting longitudinal or experimental studies to accurately measure such causal relationships. Third, the possibility of common method bias cannot be completely avoided even though we conducted a post hoc test to prevent it, because the measurement method itself targets the same source at the same time point. Thus, data must be collected from different sources when measuring the variables. Fourth, the responses for innovative work behavior, the outcome variable, were self-reported. Most studies have claimed that self-reported responses are appropriate, considering that respondents’ thoughts are reflected in those responses. However, they may be influenced by confirmation bias and social desirability. Thus, future studies must use methods that evaluate innovative behavior more accurately than self-reporting, such as supervisors’ evaluation.

## Figures and Tables

**Table 1:** Reliability analysis.

Variable		Number of Items	Cronbach’s Alpha		Items Removed
Self-leadership	Behavior-focused strategies	6	0.887	0.859	None
	Natural reward strategies	6		0.890	None
	Constructive thought pattern strategies	8		0.701	None
Innovative behavior	-	6	0.909	None	
Informal learning	Exploration of learning opportunities	5	0.926	0.900	None
	Learning with others	6		0.884	None
	Reflection	4		0.826	Items 19 and 20
Social capital	Structural social capital	7	0.887	0.821	None
	Cognitive social capital	4		0.610	Items 9 and 11
	Relational social capital	5		0.840	None

**Table 2:** Validity analysis.

Variable		Estimate	S.E.	C.R.	*p*	λ	AVE	Composite Reliability
Self-leadership	Behavior-focused strategies	0.941	0.059	15.866	***	0.773	0.756	0.902
	Natural reward strategies	1.000	-	-	-	0.671		
	Constructive thought pattern strategies	0.946	0.059	16.060	***	0.793		
Informal learning	Exploration of learning opportunities	1.000	-	-	-	0.786	0.792	0.919
	Learning with others	1.077	0.057	19.035	***	0.790		
	Reflection	0.899	0.053	17.030	***	0.707		
Innovative behavior	Innovative behavior1	0.782	0.046	16.967	***	0.685	0.660	0.920
	Innovative behavior2	0.933	0.044	21.179	***	0.809		
	Innovative behavior3	0.959	0.045	21.507	***	0.817		
	Innovative behavior4	0.895	0.040	22.395	***	0.717		
	Innovative behavior5	1.000	-	-	-	0.817		
	Innovative behavior6	0.992	0.045	22.107	***	0.834		
Social capital	Structural social capital	1.000	-	-	-	0.826	0.741	0.894
	Cognitive social capital	0.890	0.060	14.880	***	0.634		
	Relational social capital	0.940	0.056	16.833	***	0.700		

Note: N = 551. *** *p* < 0.001. χ^2^ = 258.407, Df = 80, CMIN/DF = 3.230, GFI = 0.939, AGFI = 0.909, CFI = 0.963, NFI = 0.948, IFI = 0.963, TLI = 0.951, RMSEA = 0.064, RMR = 0.029.

**Table 3:** Correlation and discriminant validity.

Variable	Mean	SD	1	2	3	4
Self-leadership	3.9410	0.52502	**(0.756)**			
Informal learning	4.0076	0.51513	0.665 **	**(0.792)**		
Innovative behavior	3.3902	0.74772	0.608 **	0.490 **	**(0.660)**	
Social capital	3.9197	0.50473	0.612 **	0.659 **	0.344 **	**(0.741)**

Note: The numbers written in bold, parentheses, and in a diagonal line are the AVE values. The numbers written below the diagonal line denote the correlation between constructs. ** *p* < 0.01.

**Table 4:** Mediating effect of informal learning.

Category	ME: Informal Learning (R-sq = 0.4461, F = 62.4799, *p* = 0.000)						DV: Innovative Behavior (R-sq = 0.4366, F = 52.4990, *p* = 0.000)					
	B	SE	t	*p*	LLCL	ULCL	B	SE	t	*p*	LLCL	ULCL
Constant	1.397	0.164	8.521	0.000	1.075	1.720	−0.388	0.256	−1.518	0.130	−0.891	0.114
Self-Leadership	0.644	0.033	19.520	0.000	0.580	0.709	0.650	0.063	10.299	0.000	0.526	0.774
Informal Learning							0.226	0.063	3.598	0.000	0.103	0.350
Sex	0.028	0.040	0.699	0.485	−0.050	0.106	−0.255	0.058	−4.372	0.000	−0.370	−0.141
Age	0.014	0.031	0.470	0.639	−0.046	0.075	0.094	0.045	2.098	0.036	0.006	0.183
Education												
Position												
Tenure	−0.004	0.016	−0.253	0.800	−0.036	0.027	0.015	0.024	0.639	0.523	−0.031	0.061
Department	−0.012	0.012	−1.026	0.306	−0.035	0.011	0.005	0.017	0.311	0.756	−0.028	0.039
Industry	−0.007	0.008	−0.859	0.391	−0.022	0.008	0.022	0.011	1.963	0.050	0.000	0.044
Employment type												
Size	0.023	0.026	0.887	0.376	−0.028	0.074	0.072	0.038	1.872	0.062	−0.004	0.147

Note: N = 551. Number of bootstrap samples = 10,000.

**Table 5:** Bootstrapping results on mediating effects of informal learning.

Indirect Effect	B	Boot SE	Boot LLCL	Boot ULCL
SL → IL → IB	0.146	0.043	0.062	0.230

Note: N = 551. Number of bootstrap samples = 10,000.

**Table 6:** Moderated mediating effect of social capital.

Category	ME: Informal Learning (R-sq = 0.4583, F = 45.6774, *p* = 0.000)						DV: Innovative Behavior (R-sq = 0.4640, F = 35.7610, *p* = 0.000)					
	B	SE	t	*p*	LLCL	ULCL	B	SE	t	*p*	LLCL	ULCL
Constant	1.490	0.182	8.195	0.000	1.133	1.848	1.665	0.930	1.789	0.074	−0.163	3.492
Self-Leadership	0.631	0.033	19.146	0.000	0.566	0.696	0.733	0.065	11.242	0.000	0.605	0.861
Informal Learning							−0.386	0.240	−1.611	0.108	−0.857	0.085
Social Capital							−0.951	0.251	−3.782	0.000	−1.445	−0.457
Informal Learning x Social Capital							0.184	0.060	3.046	0.002	0.065	0.303
Sex	−0.050	0.040	−1.243	0.215	−0.129	0.029	0.258	0.058	4.416	0.000	0.143	0.372
Age	0.018	0.033	0.543	0.587	−0.046	0.082	0.050	0.047	1.065	0.288	−0.043	0.143
Education	0.083	0.030	2.811	0.005	0.025	0.141	0.086	0.043	1.973	0.049	0.000	0.171
Position	−0.019	0.031	−0.603	0.547	−0.079	0.042	0.032	0.044	0.718	0.473	−0.055	0.119
Tenure	−0.008	0.018	−0.439	0.661	−0.042	0.027	0.030	0.026	1.181	0.238	−0.020	0.081
Department	−0.009	0.012	−0.731	0.465	−0.032	0.014	0.004	0.017	0.258	0.796	−0.029	0.038
Industry	−0.006	0.008	−0.720	0.472	−0.020	0.010	0.020	0.011	1.844	0.066	−0.001	0.042
Employment type	−0.106	0.049	−2.136	0.033	−0.203	−0.009	0.088	0.072	1.221	0.223	−0.053	0.229
Size	0.004	0.027	0.160	0.873	−0.048	0.057	0.066	0.039	1.696	0.090	−0.010	0.142

Note: N = 551. Number of bootstrap samples = 5000.

**Table 7:** Conditional indirect effects of social capital.

Index of Moderated Mediation:		Index	BootSE	BootLLCI	BootULCI
**Social capital**		0.116	0.042	0.032	0.198
Indirect effect	SC	B	Boot SE	Boot LLCL	Boot ULCL
SL → IL → IB	3.415	0.153	0.049	0.056	0.250
	3.920	0.212	0.047	0.119	0.306
	4.424	0.270	0.054	0.164	0.377

Note: N = 551. Number of bootstrap samples = 5000.

## Data Availability

Data collected and analyzed during the study are available upon reasonable request.
